# Engineered phenylalanine hydroxylase coupled with an effective cofactor synthesis and regeneration system for high-yield production of 5-hydroxytryptophan

**DOI:** 10.1186/s40643-025-00846-z

**Published:** 2025-03-06

**Authors:** Yulin Ai, Yusong Huang, Hongru Zhao, Bingmei Su, Juan Lin

**Affiliations:** 1 College of Biological Science and Engineering, Fuzhou University, Fuzhou, 350108 China; 2 Institute of Enzyme Catalysis and Synthetic Biotechnology, Fuzhou University, Fuzhou, 350108 China; 3 College of Chemical Engineering, Fuzhou University, Fuzhou, 350108 China

**Keywords:** 5-Hydroxytryptophan, Phenylalanine hydroxylase, Protein engineering, Cofactor engineering, Genome editing

## Abstract

5-Hydroxytryptophan (5-HTP) is widely used as a natural remedy for sleep disorders. In terms of biosafety, bio-derived 5-HTP is preferred over chemically synthesized 5-HTP. However, the low titer of 5-HTP in the reported microbiological methods (< 10 g/L) limits the industrialization of 5-HTP biosynthesis. In the present study, a Trp-accumulating *E. coli* strain TRP1 was constructed by blocking the degradation path (Δ*tnaA*), branching paths (Δ*pheA*, Δ*tyrA*) and repression system (Δ*trpR*, Δ*trpL*). Next, the hydroxylation module employing a phenylalanine hydroxylase mutant XcPAH^W179F^ (XC2) coupled with an MH4 regenerating system (CvPCD-EcFolM system) was screened to convert L-Trp into 5-HTP. Protein engineering was performed on hydroxylase XC2 based on the molecular dynamics simulation of the enzyme-substrate complex, and the strain TRP1-XC4 harboring the triple-mutant XcPAH^L98I/A129K/W179F^ (XC4) was able to produce 319.4 mg/L 5-HTP. Genome editing was carried out focused on accelerating product efflux (strengthening YddG) and increasing MH4 supply (strengthening FolM, FolE and FolX), resulting in a strain TRP5-XC4 to produce 13.9 g/L 5-HTP in 5 L fed-batch fermentation with a space-time yield of 0.29 g/L/h, which is the highest production and productivity record for 5-HTP biosynthesis. This study successfully provided an engineered strain and an efficient green method for the industrial synthesis of 5-HTP.

**Graphical Abstract:**

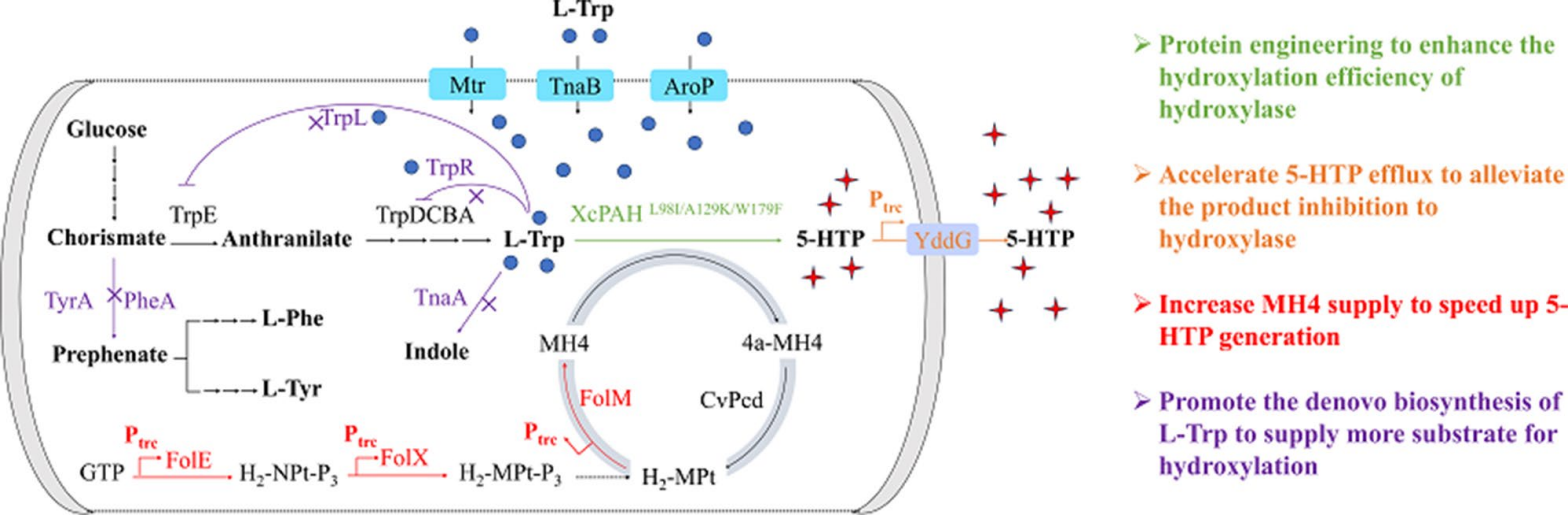

**Supplementary Information:**

The online version contains supplementary material available at 10.1186/s40643-025-00846-z.

## Introduction

5-Hydroxytryptophan (5-HTP), a derivative of tryptophan, serves as a precursor for the synthesis of the neurotransmitter 5-hydroxytryptamine (5-HT) and the amine hormone melatonin in animals (Li et al. 2025). It plays a regulatory role in various physiological functions, including emotion, behavior, sleep, pain, body temperature, and other physiological functions (Sun et al. 2024). With the rapid pace of modern life and the increasing prevalence of sleep disorders, 5-HTP has gained popularity as a natural remedy due to its non-toxic and side-effect-free properties (Birdsall 1998; Hara et al. 2013).

**Scheme 1:**
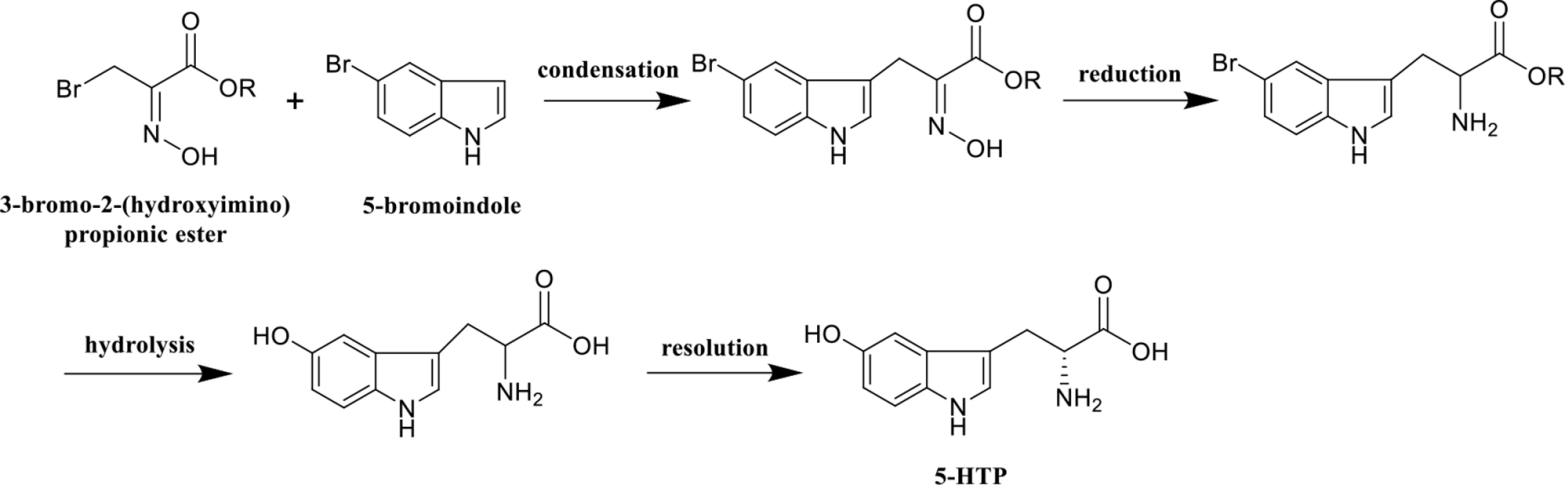
Chemical method for synthesizing 5-HTP

At present, 5-HTP is primarily extracted from the seeds of the African plant *Griffonia simplicifolia* (Lemaire et al. 2002). However, this method cannot meet the growing market demand due to the high cost and a shortage of raw materials resulting from the implementation of plant protection policies. In response to this challenge, scientists have attempted to synthesize 5-HTP through multi-step chemical reactions using 5-bromoindole and 3-bromo-2-(hydroxyimino) propionic ester as starting materials. However, the total yield was less than 30% (Fuchun et al. 2013; Liu et al. 2021) (Scheme 1).

**Scheme 2:**
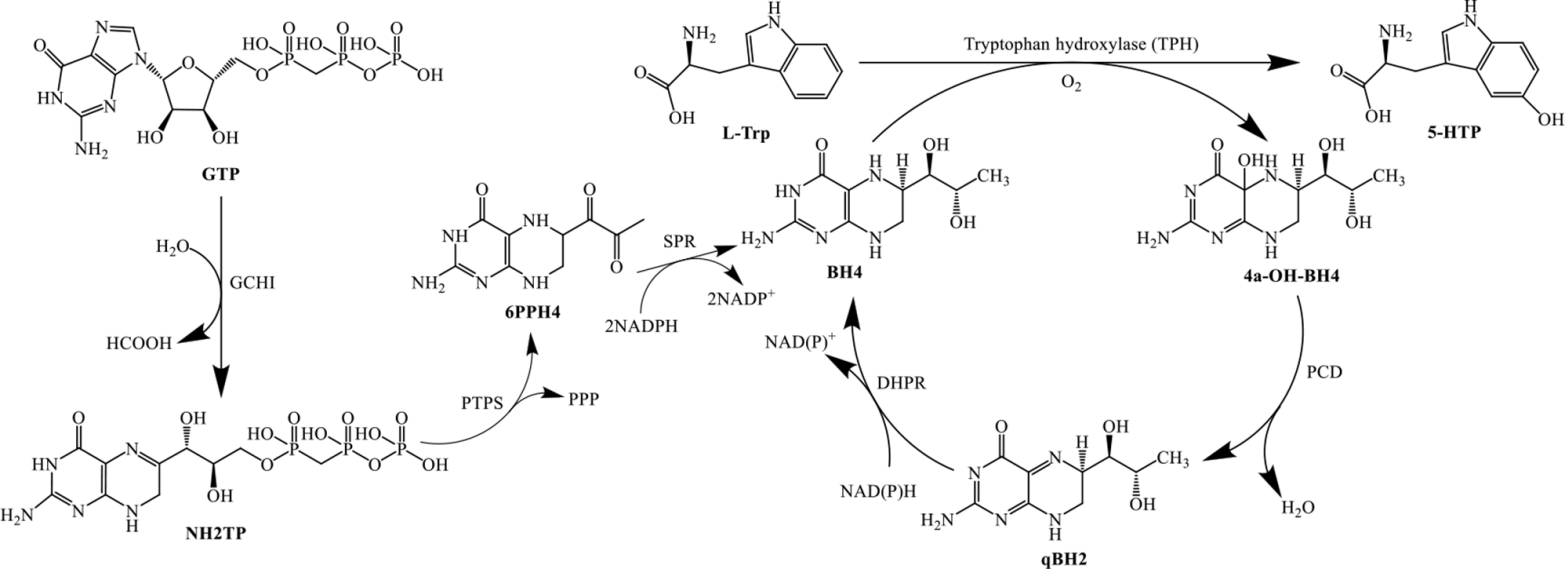
Biosynthesis path of 5-HTP with TPHs. GTP, guanosine triphosphate; GCHI, GTP cyclohydrolase I; NH2TP, 7,8-dihydroneopterin triphosphate; PTPS, 6-pyruvoyl tetrahydropterin synthase; 6PPH4, 6-pyruvoyl-5,6,7,8-tetrahydropterin; SPR, sepiapterin reductase; BH4, tetrahydrobiopterin; PCD, pterin-4α-carbinolamine dehydratase; qBH2, quinonoid dihydrobiopterin; DHPR, dihydropteridine reductas

In mammals, 5-HTP is synthesized through a hydroxylation reaction of L-Trp catalyzed by tryptophan hydroxylase (TPH). This reaction utilizes O_2_ as the oxidizing agent and requires tetrahydrobiopterin (BH4) and Fe^2+^ as cofactor and prosthetic group, respectively (Martínez et al. 2001). Based on this biosynthetic pathway, various biotransformation methods have been proposed to produce 5-HTP, which have garnered increasing attention due to the advantages, such as mild conditions, low cost, and ease of operation. During biotransformation, the engineered bacteria play a crucial role in determining 5-HTP production. The procaryotic organism *Escherichia coli* (*E. coli*) is considered an ideal host because of its well-characterized genetic information, ease of cultivation, and established gene editing methods (Wang et al. 2022). There are 3 modules required by *E. coli* to synthesize 5-HTP: hydroxylation, BH4 generation, and BH4 regeneration (Scheme 2). Moran et al. (Moran et al. 1998) found that a rabbit-derived TPH, with the N-terminal 101 amino acids and C-terminal 28 amino acids removed, can be expressed in a soluble form in *E. coli*. Wang et al. (Wang et al. 2022) overexpressed a *Schistosoma mansoni* THP and other enzymes necessary for synthesizing and regenerating BH4 in an *E. coli* strain lacking the L-Trp degradation pathway, resulting in the production of 0.93 g/L of 5-HTP after 60 h of fermentation (Table 1). Wang et al. (Wang et al. 2018) further enhanced the 5-HTP productivity of *E. coli* by optimizing the 3 modules, achieving a production of 5.1 of g/L 5-HTP in a 10 L bioreactor (Table 1). Wang and colleagues (Wang et al. 2024) semi-rationally designed a TPH from humans and obtained a thermostable variant, M30 (V275L/I412K), which produced 2.76 g/L of 5-HTP within 36 h (Table 1). Zhang et al. (Zhang et al. 2022) utilized a xylose-induced T7 RNA polymerase-P_*T7*_ promoter system to mediate the expression of functional genes, achieving the highest production of 8.58 g/L (32 h, 0.27 g/L/h space-time yield) during 5 L fermentation, aided by a NAD(P)H regeneration module and an active hydroxylation module with engineered TPH2 (Table 1).

**Scheme 3:**
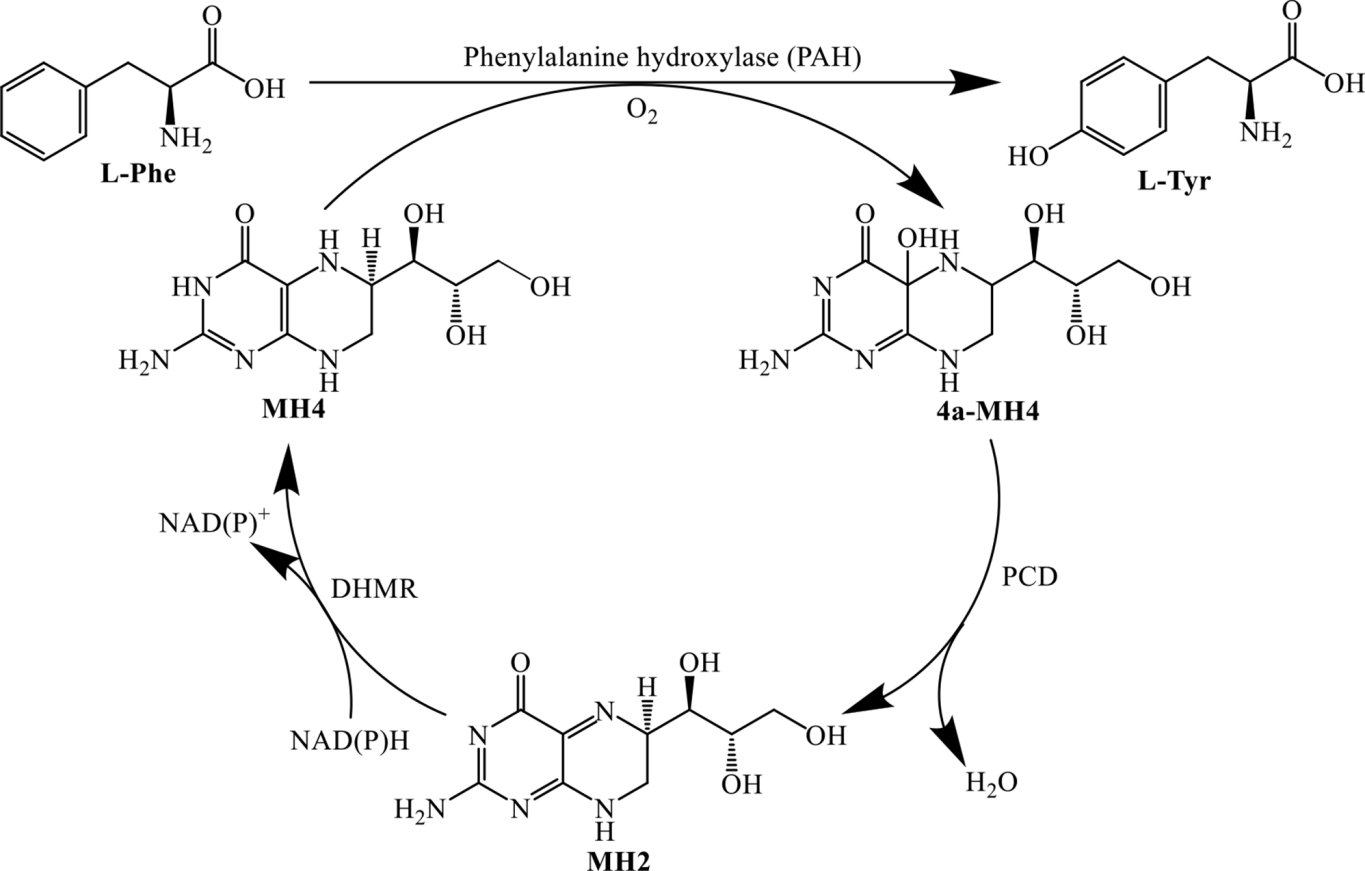
Biosynthesis path of 5-HTP with PAHs. MH4, tetrahydromonapterin; 4α-MH4, 4α-hydroxytetrahydropterin; PCD, pterin-4α-carbinolamine dehydratase; MH2, dihydromonapterin; DHMR, dihydromonapterin reductase

It has been reported that phenylalanine hydroxylases (PAHs), which belong to the family of pterin-dependent aromatic amino acid hydroxylases (AAAHs), can utilize 5,6,7,8-tetrahydromonapterin (MH4), a BH4 analogue synthesized in *E. coli*, as a cofactor for hydroxylation (Nakata et al. 1979; Pribat et al. 2010). Inspired by this funding, new cell factories were designed incorporating an engineered PAH and an MH4 regeneration module (Scheme 3, Table 1). Lin et al. (Lin et al. 2014) mutated XcPAH from *Xanthomonas campestris* based on the predicted structure, resulting in a mutant XcPAH^W179F^, which exhibited a 17.4-fold increase in activity towards L-Trp. *E. coli* strains overexpressing the mutant XcPAH^W179F^ were able to produce 1.1 g/L of 5-HTP within 16 h in conjunction with a 4a-hydroxytetrahydrobiopterin dehydratase (PCD) and a dihydromonopterin reductase (DHMR) for MH4 regeneration. Mora-Villalobos et al. (Mora-Villalobos et al. 2017) also achieved the biosynthesis of 5-HTP using a similarly engineered CtPAH from *Cupriavidus taiwanensis* mutated at the corresponding site based on the structure of XcPAH. Although there have been significant breakthroughs in the biosynthesis of 5-hydroxytryptophan (5-HTP) over the past decade, the overall yield remains quite low, with the highest recorded level being only 8.58 g/L (Zhang et al. 2022). This low yield poses challenges for industrial production. Consequently, enhancing the yield of 5-HTP is crucial for the industrial application of biological methods.

**Table 1:** Cases of 5-HTP biosynthesis in *E. coli*

AAAH	Cofactor	Strategy	5-HTP production (g/L)	Ref.
*Sm*TPH	BH4	Block of L-Trp degradation	0.93	(Wang et al. 2022)
TPH2_N∆145/C∆24_	BH4	Module optimization and protein engineering	5.10	(Wang et al. 2018)
TPH2 _N∆145/C∆24_^V275L/I412K^	BH4	Enhancement of thermostability of TPH2	2.76	(Wang et al. 2024)
TPH2_N∆145/C∆24_^E2K/N97I/P99C^	BH4	Promotor optimization, protein engineering and introduction of a NAD(P)H regeneration system	8.58	(Zhang et al. 2022)
*Xc*PAH^W179F^	MH4	Block of L-Trp degradation and protein engineering	1.10	(Lin et al. 2014)
*Ct*AAAH^W192F^	MH4	Block of L-Trp degradation and protein engineering	0.55	(Mora-Villalobos, et al. 2017)
XcPAH^W179F/L98I/A129K^	MH4	Protein enegineering, enhancement of L-Trp synthesis, block of L-Trp degradation, relief of product inhibition and promotion of MH4 generation	13.9	This study

In the present study, a Trp-accumulated *E. coli* strain was engineered and utilized as a 5-HTP producer. This strain incorporated the phenylalanine hydroxylase XcPAH from *Xanthomonas campestris*, which was rationally designed to facilitate the hydroxylation of L-Trp. Additionally, the co-expression of enzymes for MH4 regeneration was implemented. The production of 5-HTP production was further enhanced through genome editing aimed at alleviating product inhibition and promoting MH4 synthesis. The final strain, TRP5-XC4 [*E. coli* BL21(DE3) (Δ*tnaA*, Δ*pheA*, Δ*tyrA*, Δ*trpR*, Δ*trpL*, P_trc_-*yddG*, P_trc_-*folM*, P_trc_-*folE*, P_trc_-*folX*, pET30a-*xcpah*^W179F/L98I/A129K^-*cvpcd*-*ecfolM*)], achieved a production level of 13.9 g/L of 5-HTP within 48 h, resulting in a space-time yield of 0.29 g/L/h. This represents the highest level of 5-HTP biosynthesis reported at a fermentation scale.

## Experimental

### Materials

All primers used for plasmid construction, mutagenesis, and genome modification are listed in Table S2. The plasmids and strains used and constructed in this study are detailed in Tables S1 and S3, respectively. The enzymes employed for constructing hydroxylation modules are presented in Table 2. KOD One DNA polymerase, 2× Taq DNA polymerase, the Clon Express one-step cloning kit, and the FastPure Gel DNA Extraction Mini Kit were obtained from Vazyme Bio Inc. (Nanjing, China). All restriction digest enzymes were sourced from Takara Bio Inc. (Dalian, China). Rhamnose, BH4, IPTG, and standard 5-hydroxytryptophan, L-phenylalanine, L-tyrosine, and L-tryptophan were purchased from Aladdin (Shanghai, China). PCR primers were synthesized by Tsingke Bio Inc. (Beijing, China).

**Table 2:** Information of used enzymes

Enzyme	Source	Gene length(bp)	Protein length (aa)
DjTPH (Tryptophan hydroxylase)	*Dugesia japonica* (BAF79887.1)	1641	546
XcPAH (Phenylalanine hydroxylase)	*Xanthomonas campestris* (KIQ26912.1)	891	296
CtPAH (Phenylalanine hydroxylase)	*Cupriavidus taiwanensis* (SPC10285.1)	930	309
CvPCD (4a-Hydroxytetrahydrobiopterin dehydratase)	*Chromobacterium violaceum* (WP_011135913.1)	324	107
CvDpr (Nitroreductase)	*Chromobacterium violaceum* (WP_011135791.1)	654	217
EcNfsB (Nitroreductase)	*Escherichia coli* BL21 (DE3) (WP_000351487.1)	654	217
EcFolM (Dihydromonopterin reductase)	*Escherichia coli* BL21 (DE3) (HAZ7338753.1)	723	240

### CRISPR-Cas9 mediated genome editing

Genome editing of *E. coli* BL21(DE3) and its derived strains were conducted using CRISPR-Cas9 technology with the pEcCas/pTargetF system (Su et al. 2024), which was developed based on the pEcCas/pEcgRNA system (Li et al. 2021). Using this method, the genes *tnaA*, *pheA*, *tyrA*, *trpR*, and *trpL* were knocked out, and a strong promoter, P_trc_, was introduced upstream of the genes *yddG*, *folM*, *folE*, and *folX* respectively, to enhance their expression. The operational process is described using the knockout of the gene *tnnA* as an example.

The targeting plasmid for the gene *tnaA*, designated pTargetF-g-*tnaA*, was constructed using the double-primer PCR mutagenesis method with the primer pair *tnaA*-gRNA-F/*tnaA*-gRNA-R to fuse the N20 sequence into pTargetF at the upstream of the gRNA scaffold. The primers utilized are listed in Table S3. Additionally, upstream and downstream fragments (approximately 500 bp) of *tnaA* were amplified using the primer pairs *tnaA*-U-F/*tnaA*-U-R and *tnaA*-D-F/*tnaA*-D-R, respectively, with the *E. coli* BL21(DE3) genome serving as the template. Subsequently, the upstream and downstream fragments of *tnaA* were fused through overlap-extension PCR using the primer pair *tnaA*-U-F/*tnaA*-D-R, resulting in the repair template U-*tnaA*-D for knocking out *tnaA*. To insert the P_trc_ promoter, a sequence of the trc promoter was fused into the repair template between the upstream and downstream fragments.

*E. coli* BL21(DE3) cells harboring the pEcCas plasmid were induced with 10 mM L-arabinose, collected, and washed three times with 10% (v/v) glycerol to form competent cells. A total of 100 ng of the targeting plasmid pTargetF-g-*tnaA* and 400 ng repair template U-*tnaA*-D were co-transferred into 50 µL of competent cells via electroporation using a MicroPulser (BioRad, 2 mm, 2.5 kV). The cells were then resuscitated in 1 mL of LB medium at 37 °C, 200 rpm for 1 h and plated on LB agar containing kanamycin (50 mg/L) and streptomycin (50 mg/L). Transformants were verified by colony PCR using the primer pair *tnaA*-U-F/*tnaA*-D-R and confirmed through DNA sequencing. The positive colony, *E. coli* BL21(DE3) (Δ*tnaA*, pTargetF-g-*tnaA*, pEcCas), was cultivated in LB medium supplemented with 10 mM rhamnose to eliminate the targeting plasmid pTargetF-g-*tnaA* and screened on LB plates containing kanamycin (50 mg/L). The colony sensitive to streptomycin, identified as *E. coli* BL21(DE3) (Δ*tnaA*, pEcCas), was further cultivated in LB medium supplemented with 5 g/L glucose to eliminate the plasmid pEcCas and screened on LB plate supplemented with 5 g/L glucose and 10 g/L sucrose. The colony that was sensitive to kanamycin was identified as the strain *E. coli* BL21(DE3) (Δ*tnaA*). Strain *E. coli* BL21(DE3) (Δ*tnaA*, pEcCas) was prepared as competent cells for a new round of genome editing.

### Construction of recombinant plasmids

The method for constructing the recombinant plasmids cantaining multiple genes in the polycistronic pET30a vector is described taking the construction of the plasmid pET30a-*xcpah*-*cvpcd*-*ecfolm* (pXC1) as an example (Su et al. 2024). The genes *xcpah*, *cvpcd*, and *ecfolM* were amplified using the corresponding primer pairs XC-F/XC-CV-R, XC-CV-F/CV-EC-R, and CV-EC-F/EC-R, with synthesized genes serving as templates. The fusion fragment *xcpah*-*cvpcd*-*ecfolm* was amplified through overlap-extension PCR using the primer pair XC-F/EC-R, with purified *xcpah*, *cvpcd*, and *ecfolm* fragments as templates. Following double digestion with BamHI and XhoI, the *xcpah*-*cvpcd*-*ecfolm* fragment was recombined into pET30a under the control of a T7 promoter using T4 ligase. The recombinant plasmid was then introduced into competent TRP1 cells, resulting in the strain TRP1-XC1. Using the same method, the strains TRP1-CT1 (harboring *ctpah-cvpcd-ecfolm* fragment), TRP1-DJ1 (harboring the *djtph-cvpcd-cvdpr* fragment), and TRP1-DJ2 (harboring the *djtph-cvpcd-ecnfsb* fragment) were constructed.

### HTP production by shake-flask fermentation

A single colony of engineered bacteria was inoculated in 25 mL of LB medium (10 g/L tryptone, 5 g/L yeast extract, and 10 g/L NaCl) supplemented with kanamycin (50 mg/L) and incubated at 37 °C, 200 rpm for 12 h to prepare the initial seed culture. Subsequently, 2.5 mL of the seed culture was inoculated into a 250 mL Erlenmeyer flask containing 25 mL kanamycin-contained M9Y medium (10 g/L glucose, 6 g/L Na_2_HPO_4_, 0.5 g/L NaCl, 3 g/L KH_2_PO_4_, 1 g/L NH_4_Cl, 2 g/L yeast extract, 2 g/L sodium citrate dihydrate, 246.5 mg/L MgSO_4_·7H_2_O, 14.7 mg/L CaCl_2_·2H_2_O, 27.8 mg/L FeSO_4_·7H_2_O, pH7.2) and grown at 37 °C, 200 rpm. When the OD_600_ reached 2.5, 0.1 mM IPTG was added to induce the expression of the introduced enzymes and initiate hydroxylation at 25 °C, 200 rpm for 24 h. To analyze the hydroxylation capacity of the strains with respect to their nature substrates, 10 µM BH4 and 10 mM L-Trp were added to the TPH group, while 10 mM L-Phe was added to the PAH group. In the shake-flask aimed at converting L-Trp into 5-HTP using the PAH system, 10 mM L-Trp was added as the substrate. During the cultivation process, 25% ammonium hydroxide was used to maintain the culture pH at approximately 7.0.

### Component analysis of fermentation broth

After fermentation, OD_600_ was measured to assess cell growth. The cells were removed by centrifugation (12000 rpm, 1 min). The supernatant was appropriately diluted for residual sugar detection using the DNS method (Li et al. 2011) and for product quantification via HPLC. The supernatant was diluted with a water phase (a water solution adjusted to pH3.4 with acetic acid) and filtrated using a 0.22 μm hydrophilic membrane. A 20 µL sample was loaded onto a Supersil ODS2 C18 column (Elite, China) for detection at 35 °C, utilizing an acetonitrile/water phase (6:94 v/v) as the mobile phase at a flow rate of 1 mL/min. 5-HTP, L-Tyr, and L-Trp were monitored using an ultraviolet detector at a wavelength of 276 nm, while L-Phe was detected at 196 nm.

### Molecular dynamics simulation for enzyme-substrate complexes

The apoenzyme structure of hydroxylase (XC1 or its mutant) was modeled using the crystal structure of phenylalanine hydroxylase CvPAH from *Chromobacterium violaceum* (PDB id: 3TK2 (Ronau et al. 2013) as the template. Based on the binding modes of BH4 and Fe^2+^ ion in the crystal structure of human phenylalanine hydroxylase HsPAH (PDB id: 1MMK (Andersen et al. 2003), MH4 and Fe^2+^ ion were positioned in the corresponding regions of hydroxylase. The micro molecule MH4 was standardized using the Antechamber tool in Amber20 (Su et al. 2024). Subsequently, the coordinate and topology files for holoenzymes (XC1-MH4, XC2-MH4, or XC4-MH4) were generated after solvation with TIP3PBOX water and neutralization with Na^+^ in the LEaP module. These files were then utilized for energy minimization using the pmemd.cuda program (Case et al. 2023; Su et al. 2019). The resulting models of the holoenzymes were evaluated for accuracy in the SAVES server.

L-Trp was docked into the holoenzyme within a cube measuring 40 Å × 40 Å × 40 Å, centered on the Fe^2+^ ion. From a total of 100 conformations, the conformation of L-Trp that orientated its C5’ (specifically C7_sub_) within 4 Å of the Fe^2+^ ion [d(FE-C7_sub_)] was selected to form an enzyme-substrate complex with the holoenzyme and subsequently minimized using AMBER 20.

MD simulations were conducted as previously reported (Su et al. 2022).The system underwent a series of steps: 50 ps of NVT heating (ramping from 0 to 300 K), followed by 50 ps of protein-restricted NVT equilibration, 500 ps of protein-unrestricted NPT equilibration, and finally, 20 ns of NPT simulation for trajectory production. To illustrate the binding mode, the frame with the lowest total energy was selected as representative of the enzyme-substrate complex in the MD simulation. The catalytic distances, d(FE-C7_sub_) and d(FE-C2_MH4_), were calculated using the cpptraj program.

### Sequence conservatism analysis

The sequence conservatism of XcPAH was analyzed using the Phyre2 server (Kelley et al. 2015) and visualized with the Weblogo tool (Crooks et al. 2004).

### Mutagenesis

Using the plasmid pXC1 as a template, site-directed mutagenesis was performed using the double-primer PCR mutagenesis method with mutagenic primers (listed in Table S1) according to the manufacturer’s instructions for KOD One. The PCR product (2 µL) was digested with the dpnI enzyme (37 °C, 2 h) and then transformed into 60 µL of TRP1 competent cells. The transformants, which were verified by DNA sequencing, were cultivated for 5-HTP production in shake-flask fermentation.

### Biotransformation in 5 L bioreactor for 5-HTP production

The single colony of strain TRP5-XC4, resuscitated on an LB agar plate supplemented with kanamycin (50 mg/L), was transferred into 75 mL of kanamycin-containing LB medium and cultured at 37 °C and 200 rpm for 12 h. Subsequently, the seed culture was inoculated into a 5 L bioreactor containing 3 L of fermentation medium, which consisted of 10 g/L glycerol, 15 g/L yeast extract, 4 g/L K_2_HPO_4_, 2.24 g/L NaH_2_PO_4_, 3 g/L NaCl, 2.5 g/L (NH_4_)_2_SO_4_·7H_2_O, 2.1 g/L citric acid monohydrate, 0.5 g/L MgSO_4_·7H_2_O, 0.1 g/L FeSO_4_·7H_2_O, 0.01 g/L CuSO_4_·7H_2_O, 0.005 g/L MnSO_4_·H_2_O, 0.02 g/L ZnSO_4_, 0.002 g/L V_B1_, 1 g/L L-Tyr and 1 g/L L-Phe to initiate fermentation. The pH was maintained at 7.0 through the automatic addition of NH_4_OH (25%, v/v). The dissolved oxygen (DO) level was kept at approximately 30% by adjusting the stirrer speed (maximum limit: 600 rpm). During the early stages of fermentation, the temperature was held at 37 °C. When the feed signal, indicated by a rapid increase in pH and DO values, was detected, a glucose solution (600 g/L) was added to maintain the sugar concentration in the culture at 0.1–2 g/L, and 1 g of lactose was introduced every 5 h to induce the expression of functional genes. Concurrently, the temperature was lowered to 25 °C. As feeding commenced, 5 g of L-Trp was added as the substrate for 5-HTP production. Subsequently, L-Trp was continuously added at a rate of 0.5 g/h. Throughout the process, residual glucose, 5-HTP titer, L-Trp and wet cell weight were measured at intervals of 3–4 h. The conversion of exogenous L-Trp was calculated according to the equation as followed:

$$\:Conv.=\frac{{C}_{5-HTP}\times\:V}{{m}_{L-Trp}-{C}_{L-Trp}\times\:V}\times\:100\%$$

$$\:{C}_{5-HTP}$$: 5-HTP titer, g/L;

V: Volume of fermentation broth;

$\:{m}_{L-Trp}$: Exogenously added L-Trp;

$\:{C}_{L-Trp}$: L-Trp titer, g/Lss

## Results and discussion

### Construction of trp*-*accumulating *E. coli* strain

**Fig. 1:**
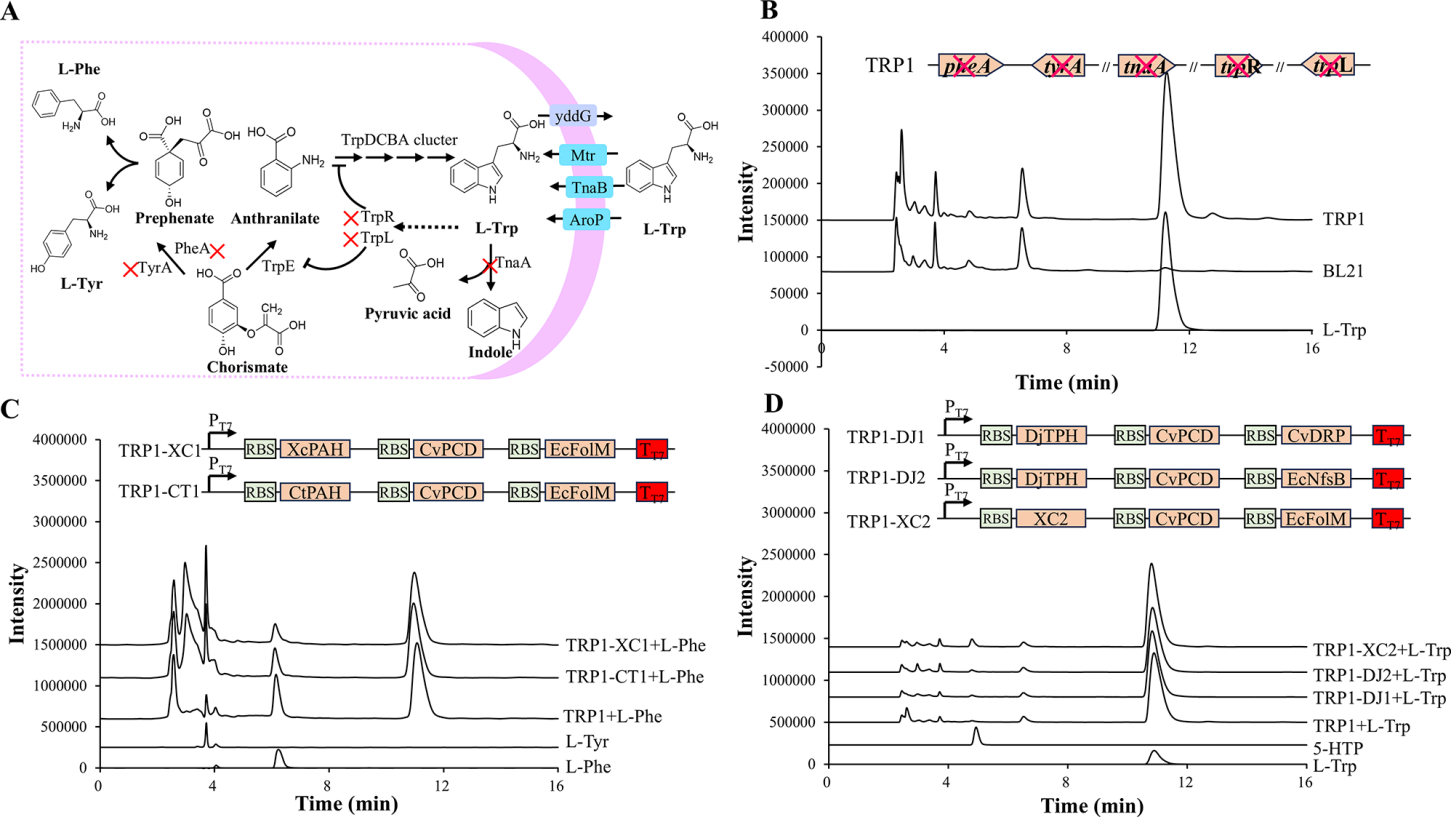
Construction of 5-HTP-producing strains. (**A**) “Increasing income and reducing expenditure” strategy to accumulate L-Trp in strain TRP1; (**B**) HPLC analysis of strain TRP1; (**C**) Hydroxylation performance of strains towards L-Phe; (**D**) Hydroxylation performance of strains towards L-Trp

To prevent the degradation of L-Trp and enhanced its availability for bioconversion into 5-HTP, a strategy of “increasing income and reducing expenditure” was employed to engineer *E. coli* BL21 (DE3) for L-Trp accumulation, as illustrated in the metabolic network (Fig. 1A) (Ikeda 2006). L-Trp is an endogenous amino acid utilized as a substrate for protein expression and the production of Trp derivatives in mammalian cells. However, L-Trp is rapidly consumed by *E. coli* as soon as it is generated, primarily due to the degradation pathway initiated by tryptophanase, which is encoded by the gene *tnaA*. Additionally, L-Trp biosynthesis shares its precursor, chorismite, with 2 branching pathways: L-Phe biosynthesis and L-Tyr biosynthesis. Consequently, disrupting these branching pathways was predicted to be a feasible approach to increase carbon flux for L-Trp biosynthesis. Chorismate is converted into prephenate by chorismate mutase, which is encoded by the genes *pheA* and *tyrA* in *E. coli*. Furthermore, the biosynthesis of L-Trp is repressed by a repressor and an attenuator encoded by the genes *trpR* and *trpL.* To construct an L-Trp accumulating strain of *E. coli*, the genes *tnaA*,* pheA*, *tyrA*,* trpR*, and *trpL* were knocked out using the CRISP-Cas9 method with the pEcCas/pTargetF system (Su et al. 2024), resulting in the generation of strain TRP1 [*E. coli* BL21(DE3) (Δ*tnaA*, Δ*pheA*, Δ*tyrA*, Δ*trpR*, Δ*trpL*)]. Strain TRP1 was cultivated in shake-flask fermentation and accumulated 143.32 mg/L of L-Trp in the culture after 24 h (Fig. 1B). Strain TRP1 served as the initial chassis for the subsequent biosynthesis of 5-HTP using a heterologous hydroxylation module, supported by an effective cofactor regeneration system.

### Screen of hydroxylation module and the corresponding cofactor regeneration module

To produce 5-HTP through the bioconversion of L-Trp in strain TRP1, various aromatic amino acid hydroxylases (AAAHs) were overexpressed in conjunction with enzymes for cofactor regeneration (Table 2). For tryptophan hydroxylase (TPH), DjTPH from *Dugesia japonica* was utilized for hydroxylation, while tetrahydrobiopterin (BH4) was regenerated using 2 enzyme systems: a 4a-hydroxytetrahydrobiopterin dehydratase and a nitroreductase sourced from different organisms (the CvPCD-EcNfsB system and the CvPCD-CvDPR system). This process resulted in the generation of 2 strains, TRP1-DJ1 and TRP1-DJ2 (Table S3). For phenylalanine hydroxylase (PAH), XcPAH and CtPAH from *Xanthomonas campestris* and *Cupriavidus taiwanensis*, respectively, were employed for hydroxylation, with MH4 being regenerated through an enzyme system consisting of a 4a-hydroxytetrahydrobiopterin dehydratase and a dihydromonapterin reductase (the CvPCD-EcFolM system). This led to the creation of 2 strains, TRP1-XC1 and TRP1-CT1 (Table S3).

All constructed strains were cultivated and induced with 0.1 mM IPTG. The hydroxylation activity of the strains was analyzed by detecting the hydroxylated products from their nature substrates (10 µM BH4 and 10 mM L-Trp were supplemented for the TPH group, while 10 mM L-Phe was supplemented for the PAH group) using HPLC. As shown in Fig. 1C, the stains in the PAH group (TRP1-XC1 and TRP1-CT1) were capable of converting L-Phe into L-Tyr. Comparatively, strain TRP1-XC1 exhibited higher hydroxylation activity than strain TRP1-CT1, as evidenced by the greater L-Tyr production from stain TRP1-XC1 compared to TRP1-CT1 (329 mg/L vs. 223 mg/L). In contrast, the stains in the TPH group (TRP1-DJ1 and TRP1-DJ2) were unable to convert L-Trp into 5-HTP (Fig. 1D). The primary reason for the inactivity of TRP1-DJ1 and TRP1-DJ2 was identified as their inability to express DjTPH in a soluble form (Figure S1).

The substrate selectivity of XcPAH towards L-Phe was modified to L-Trp as reported by Lin (Lin et al. 2014). The W179 site of XcPAH was substituted with Phe using a double-primer PCR mutagenesis method, with plasmid pXC1 serving as the template. The resulting plasmid, pXC2, was introduced into strain TRP1, creating stain TRP1-XC2. Strain TRP1-XC2 demonstrated the ability to produce 190 mg/L of 5-HTP during the shake-flask fermentation using L-Trp as the substrate, indicating that an initial 5-HTP-producing strain was successfully constructed (Fig. 1D).

### Protein engineering of XcPAH to enhance the 5-HTP production

The hydroxylation reaction mediated by the engineered phenylalanine hydroxylase XcPAH was the rate-limiting step in 5-HTP biosynthesis. To enhance 5-HTP production, mutagenesis was conducted using XC2 (XcPAH^W179F^) as the parent, and the resulting mutants were expressed in the TRP1strain for functional comparison.

**Fig. 2:**
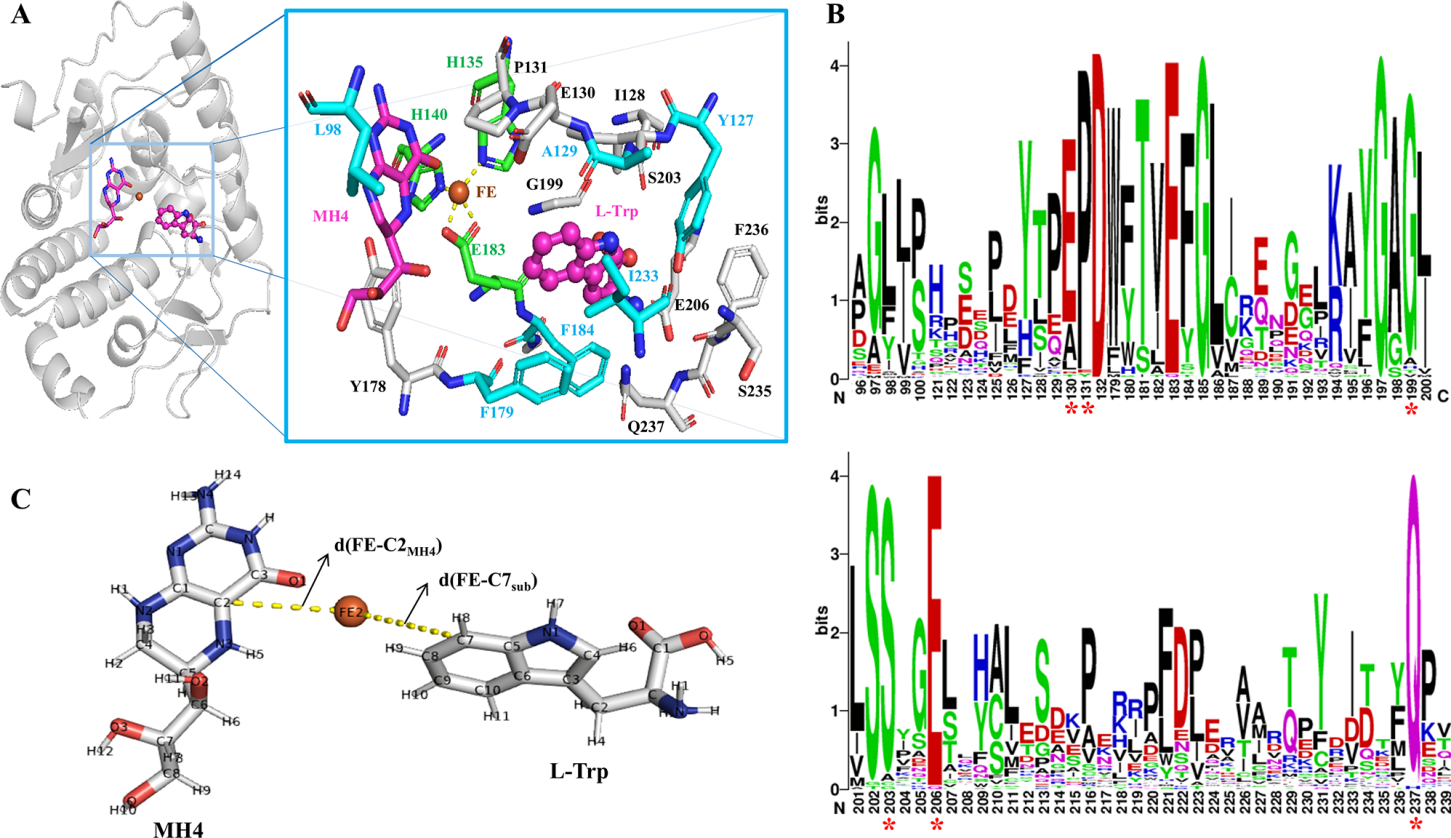
Analysis of the structure and key sites of XcPAH. (**A**) Catalytic center of the XcPAH complexed with MH4 and L-Trp; (**B**) Conserved analysis of candidate sites for engineering; (**C**) Catalytic distances for hydroxylation

The enzyme-substrate complex XC2-TRP was constructed using AutoDock 4 and subsequently energy minimized with AMBER 20. As illustrated in Fig. 2A, the Fe^2+^ ion was stabilized by the active site residues H135, H140, and E183. Meanwhile, the substrate L-Trp was surrounded by 16 residues, among which the residues E130, P131, G199, S203, E206, and Q237 were predicted to be conserved (Fig. 2B). Considering the side-chain orientation and the conservation of these residues, 6 sites (L98, Y127, A129, F179, F184, and I233) were selected as targets for mutagenesis.

Taking XC2 as the parent, L98 and I233 were mutated for different reasons. At site L98, it was substituted with similar residues (Ile and Val) or aromatic residues (Tyr and Phe) to attract the indole group of L-Trp. For site I233, a smaller hydrophobic residue (Ala or Val) was predicted to create less steric hindrance, facilitating the accommodation of the substrate L-Trp. Among the 6 dual mutants, XcPAH^W179F/L98I^ (XC3) was identified as the most effective, enabling strain TRP1 to achieve the highest production of 5-HTP (270.3 mg/L, Table 3).

**Table 3:** 5-HTP production of engineering strain after mutagenesis

Enzyme for hydroxylation in strain TRP1	5-HTP production (mg/L)
XcPAH (XC1)	0
XcPAH^W179I^	0
XcPAH^W179L^	0
XcPAH^W179M^	0
XcPAH^W179Y^	122 ± 6
**XcPAH** ^**W179F**^ **(XC2)**	**190 ± 13**
XcPAH^W179F/L98V^	96.1 ± 1.2
XcPAH^W179F/L98Y^	117.4 ± 0.9
XcPAH^W179F/L98F^	155.0 ± 1.9
CtPAH^W179F^	113 ± 5
DjTPH (DJ1)	0
DjTPH (DJ2)	0
**XcPAH** ^**W179F/L98I**^ **(XC3)**	**270.3 ± 2.2**
XcPAH^W179F/I233V^	177.60 ± 0.31
XcPAH^W179F/I233A^	146 ± 7
XcPAH^W179F/L98I/Y127H^	234.3 ± 2.5
XcPAH^W179F/L98I/Y127F^	253 ± 13
XcPAH^W179F/L98I/F184Y^	263 ± 15
XcPAH^W179F/L98I/A129T^	222.5 ± 3.1
XcPAH^W179F/L98I/A129G^	256 ± 8
XcPAH^W179F/L98I/A129N^	258.5 ± 3.7
**XcPAH** ^**W179F/L98I/A129K**^ **(XC4)**	**319 ± 19**

**Fig. 3:**
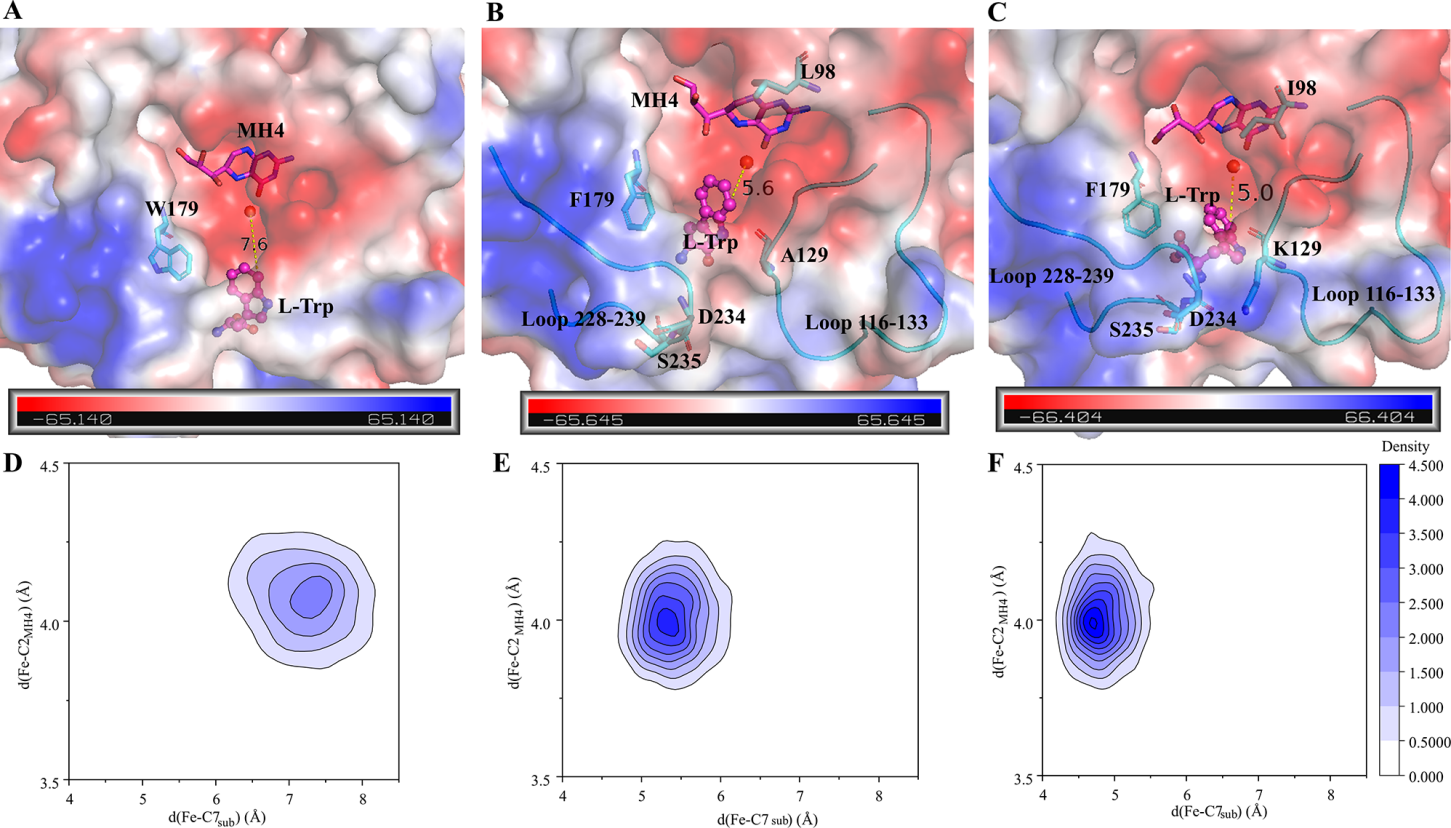
Comparison of the binding modes and catalytic distances between complexes. (**A**) Binding mode of L-Trp in XC1; (**B**) Binding mode of L-Trp in XC2; (**C**) Binding mode of L-Trp in XC4; (**D**) Catalytic distances in the complex XC1-L-Trp throughout the entire MD simulation; (**E**) Catalytic distances in the complex XC2-L-Trp throughout the entire MD simulation; (**F**) Catalytic distances in the complex XC4-L-Trp throughout the entire MD simulation

Taking XC3 as the new parent, Y127 and F184 were mutated based on their conservation (Fig. 2A). The A129 site is located on Loop 116–133 and is positioned at the polar entrance of the substrate binding pocket (Fig. 3B). Additionally, site A129 orients its side chain towards the solvent and is in close proximity to the polar region of the adjacent Loop 228–239. Consequently, site A129 was substituted with residues of various polarity, including Gly, Thr, Gln, and Lys. Following shake-flask fermentation, a triple mutant, XcPAH^W179F/L98I/A129K^ (designated as XC4), was identified as having the highest hydroxylation performance, enabling strain TRP1 to produce 319 mg/L of 5-HTP, which was 1.68 times the amount produced by strain TRP1-XC2 (Table 3).

### Structural mechanism for the property change after mutation

#### Site 179 regulates the substrate selectivity

Site W179 is located at the entrance of the substrate binding pocket of XcPAH. During the evolutionary process of aromatic amino acid hydroxylases (AAAHs), this site has a preference for hydrophobic residues such as Trp or Phe (Fig. 2B). It has been observed that this site favors Trp in phenylalanine hydroxylases, while Phe is more commonly preferred by tryptophan hydroxylases. Inspired by these findings, the W179F mutant of XcPAH was constructed, successfully altering the substrate selectivity from L-Phe to L-Trp in this study, as well as in other literature reported by multiple research teams.

As shown in Fig. 3A, in the wild-type XcPAH, the bulky side chain of W179 prevents the substrate L-Trp from approaching the prosthetic group Fe^2+^ ion, resulting in a long catalytic distance d(FE-C7_sub_) (Figs. 2C and 3D), which hinders hydroxylation. In contrast, after the W179F mutation, the bulky indole ring is replaced with a smaller benzene ring at position 179, creating more space for the substrate L-Trp to approach the active center. This is illustrated by the representative structure of the XC2-L-Trp complex (Fig. 3B) and the shorter catalytic distance d(FE-C7_sub_) compared to that of the XC1-L-Trp complex (Fig. 3D and E) during the MD simulation. When W179 is substituted with another aromatic residue, Tyr, 122 mg/L of 5-HTP is produced. However, when W179 is replaced with aliphatic hydrophobic residues such as Ile, Leu, and Met, no 5-HTP is generated from L-Trp. These results indicate that in XcPAH or its variants, an aromatic ring at position 179 is essential for the binding, stabilization, and even hydroxylation of the aromatic substrates L-Trp or L-Phe. Due to its critical spatial position, the size of the side chain determines the substrate selectivity of the enzyme. A bulky dicyclic residue, Trp, at position 179 dictates the role of XcPAH as a phenylalanine hydroxylase, while a monocyclic residue, Phe, at this site determines its role as a tryptophan hydroxylase.

#### L98I/A129K mutation reshaped the substrate binding pocket and further enhanced the hydroxylation activity of XC1

At position 98, hydrophobic aromatic residues (Tyr and Phe) were not beneficial to the activity of XcPAH towards the target substrate L-Trp, as evidenced by the decreased production of 5-HTP in the XcPAH^W179F/L98Y^ and XcPAH^W179F/L98F^ variants (Table 3). This indicates that a hydrophobic residue with appropriate flexibility is crucial for this site to ensure the hydroxylation function of XcPAH. When Leu at site 98 was replaced with Ile, an isomer that differs in the position of methyl substitution, 5-HTP production increased by 42%. Site 98 is located at the entrance of the cavity that accommodates the cofactor MH4, and its side chain plays a key role in stabilizing MH4 during the transfer of oxygen between the oxygen molecule, MH4, and Fe^2+^ ion. The higher production of 5-HTP in the XcPAH^W179F/L98I^ (XC3) variant compared to XC2 indicates that Ile is more suitable than Leu at this site for stabilizing MH4.

It was predicted that the interaction between the secondary structures of Loop 116–133 and Loop 228–239 is crucial for regulating the conformational change of the catalytic center and maintaining stability during the reaction. Prior to mutagenesis, these two loops may have been stabilized by the hydrophobic interactions between A129 and I233, as well as the hydrogen bonds formed by Y127, D234, and S235. When A129 was replaced with the alkaline residue Lys, the polarity of the interface between the 2 loops changed, resulting in a stable conformation with decreased fluctuations, as quantified by the root mean square fluctuation (RMSF) (Figure S2).

In summary, the three-point mutation L98I/A129K/W179F has reshaped the structure of XC4, creating a suitable substrate binding pocket and a stable catalytic center that stabilizes the cofactor, accommodates L-Trp, and promotes its binding at the catalytic center with a short catalytic distance (Fig. 3F). This configuration favors the hydroxylation of L-Trp.

### Genome editing for further enhancing 5-HTP production

#### Relieve production inhibition by strengthening the efflux pump

During the bioconversion of L-Trp into 5-HTP, it was anticipated that 5-HTP would be secreted into the fermentation broth for 2 primary reasons. First, it is easier to isolate 5-HTP from the fermentation broth than from the cells at a factory scale. Second, high levels of intracellular end products have been reported to inhibit the enzymes of the biosynthetic pathway, slow down the reactions, and, in some cases, inhibit growth. This can lead to instability of the strain and create challenges when scaling up fermentation processes (Hoek et al. 2020; Yang et al. 2021). Therefore, promoting the efflux of 5-HTP is crucial for the sustainable production of this compound.

The efflux pump YddG is responsible for the export of aromatic amino acids (L-Phe, L-Trp, and L-Tyr) (Doroshenko et al. 2007; Liu et al. 2012; Wang et al. 2013). The target product, 5-HTP, is a derivative of L-Trp, differing only in the substituent group at the C5’ position. With this in mind, YddG was predicted to be capable of promoting the export of 5-HTP. To verify this hypothesis, a strong promoter, P_trc_, was introduced before the ribosome binding site (RBS) of the *yddG* gene to enhance its transcription, resulting in the creation of a new engineered strain, TRP2 (TRP1, P_trc_-*yddG*). The recombinant plasmid, pXC4, which contains the genes encoding XC4, CvPCD, and EcFolM, was then transferred into strain TRP2, yielding a new 5-HTP-producing stain, TRP2-XC4.

**Fig. 4:**
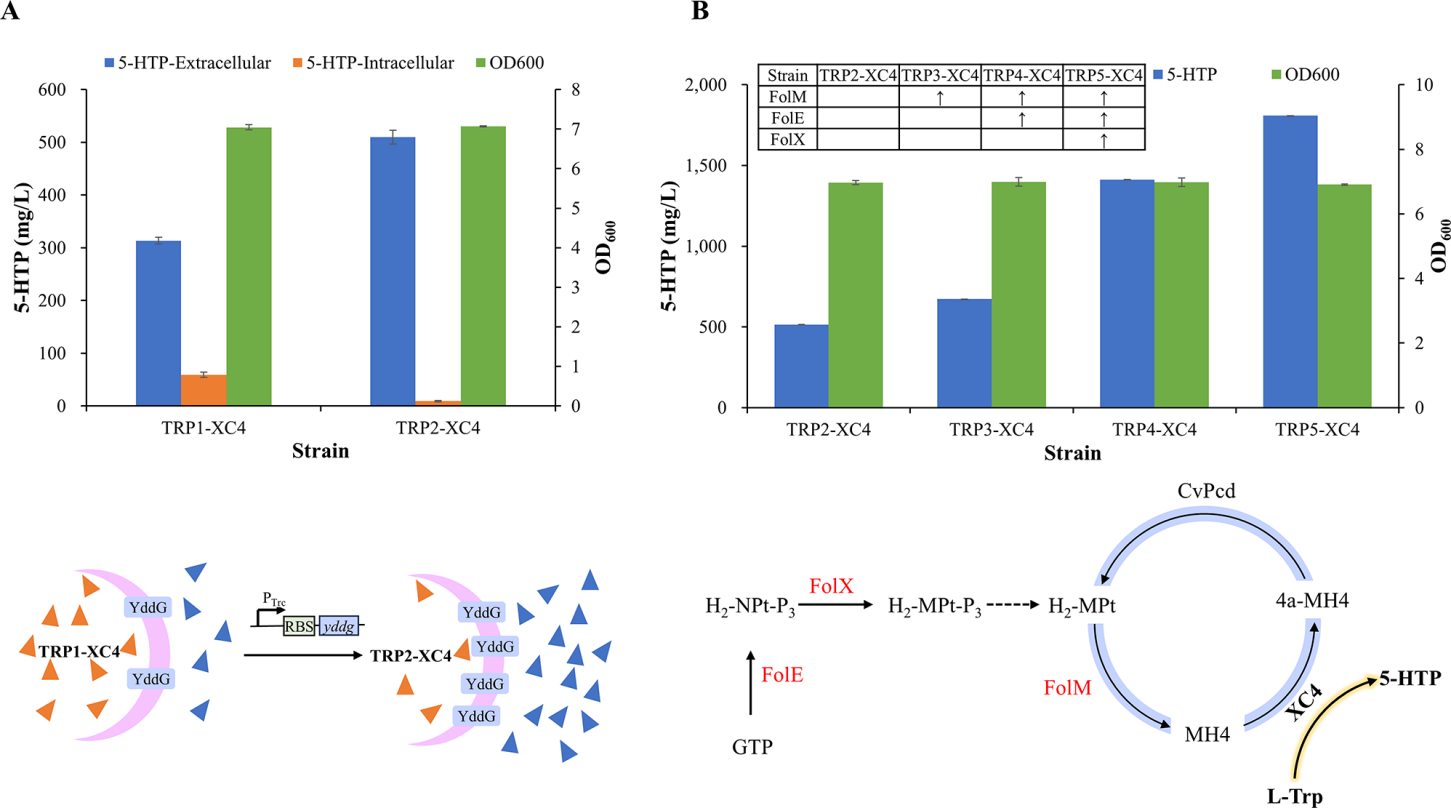
Genome editing to enhance 5-HTP production. (**A**) Strengthening the efflux pump YddG; (**B**) Cofactor engineering

Strain TRP2-XC4 was cultured for the production of 5-HTP in shake-flask fermentation using 10 mM L-Trp as the substrate and was compared with strain TRP1-XC4. As shown in Fig. 4A, the high-level expression of the efflux pump YddG did not affect cell growth but significantly accelerated the production of 5-HTP. After 24 h of cultivation, strain TRP2-XC4 produced 518.9 mg/L of 5-HTP, which was 1.39 times the production of strain TRP1-XC4 (372.5 mg/L).

As expected, the intracellular concentration of 5-HTP in strain TRP2-XC4 was significantly lower than that in strain TRP1-XC4 (9.1 vs. 59 mg/L). Conversely, the extracellular concentration of 5-HTP in strain TRP2-XC4 was 62.6% higher than that in strain TRP1-XC4 (510 vs. 313 mg/L). These results suggest that the YddG pump, which is responsible for the export of aromatic amino acids, is also capable of exporting 5-HTP. Enhancing the expression of the *yddG* gene can reduce the intracellular accumulation of 5-HTP through efflux, thereby alleviating the inhibitory effect of 5-HTP on the hydroxylase XC4 in cells. This process significantly promotes the production of 5-HTP.

#### Accelerate the 5-HTP production by cofactor engineering

Hydroxylase XcPAH and its variants can utilize MH4 as a cofactor for oxygen transfer instead of BH4. It has been reported that MH4 can be synthesized endogenously from GTP by *E. coli* through a series of enzymatic reactions mediated by FolE, FolX, FolM, FolB, PhoA, PhoB, and FolQ (Pribat et al. 2010) **(Figure S3)**. However, the amount of MH4 produced is sufficient only for normal growth and does not meet the demands of overexpressed hydroxylase, which limits the hydroxylation performance of the cells.

MH4 is converted into pterin-4a-carbinolamine (4a-MH4) following the hydroxylation of L-Trp, a process catalyzed by XC4. To enhance the utilization rate of MH4 in the cell factory, the enzyme 4a-hydroxytetrahydrobiopterin dehydratase (CvPCD) was introduced to convert 4a-MH4 into H_2_-Mpt, which is subsequently reduced to MH4 by FolM (Fig. 4B). A strong promoter, P_trc_, was incorporated to boost the expression of FolM. The resulting strain, TRP3-XC4 (strain TRP3 harboring plasmid pXC4), produced 673 mg/L of 5-HTP after 24 h of cultivation, representing a 30.6% increase compared to strain TRP2-XC4 (Fig. 4B).

MH4 is synthesized using the endogenous GTP as the raw material in *E. coli* cells (**Figure S3**). The initial reaction is catalyzed by FolE (GTP cyclohydrolase IA), producing H_2_-NPt-P_3_, which is then epimerized to H_2_-MPt-P_3_ by FolX (D-erythro-7,8-dihydroneopterin triphosphate epimerase). Consequently, FolE and FolX are considered the rate-limiting enzymes in the synthesis of MH4. As illustrated in Fig. 4B, the production of 5-HTP in strain TRP4-XC4, an engineered strain with enhanced expression of FolE compared to strain TRP3-XC4, increased significantly to 1.41 g/L. Furthermore, 5-HTP production was further improved to 1.81 g/L when the expression of 3 cofactor-related enzymes (FolM, FolE, and FolX) was simultaneously enhanced in strain TRP5-XC4.

During the cofactor engineering process, the OD_600_ values remained relatively constant among the strains TRP2-XC4, TRP3-XC4, TRP4-XC4, and TRP5-XC4, indicating that the regulation of MH4 synthesis did not impact cell growth. We were excited to find that the cofactor engineering strategy enhanced 5-HTP production by 251.1% (1.81 g/L for strains TRP5-XC4 compared to 0.51 g/L for strain TRP2-XC4), demonstrating that increasing the cofactor supply is an effective means of accelerating the enzymatic process.

### High-titer production of 5-HTP through efficient biotransformation of L-Trp in a 5-L bioreactor

The TRP5-XC4 strain, recognized as the most efficient producer of 5-HTP, was developed through a combination of protein engineering and genomic regulation. This strain was employed for 5-HTP production in a 5-L biotransformation process using L-Trp as the substrate.

**Fig. 5:**
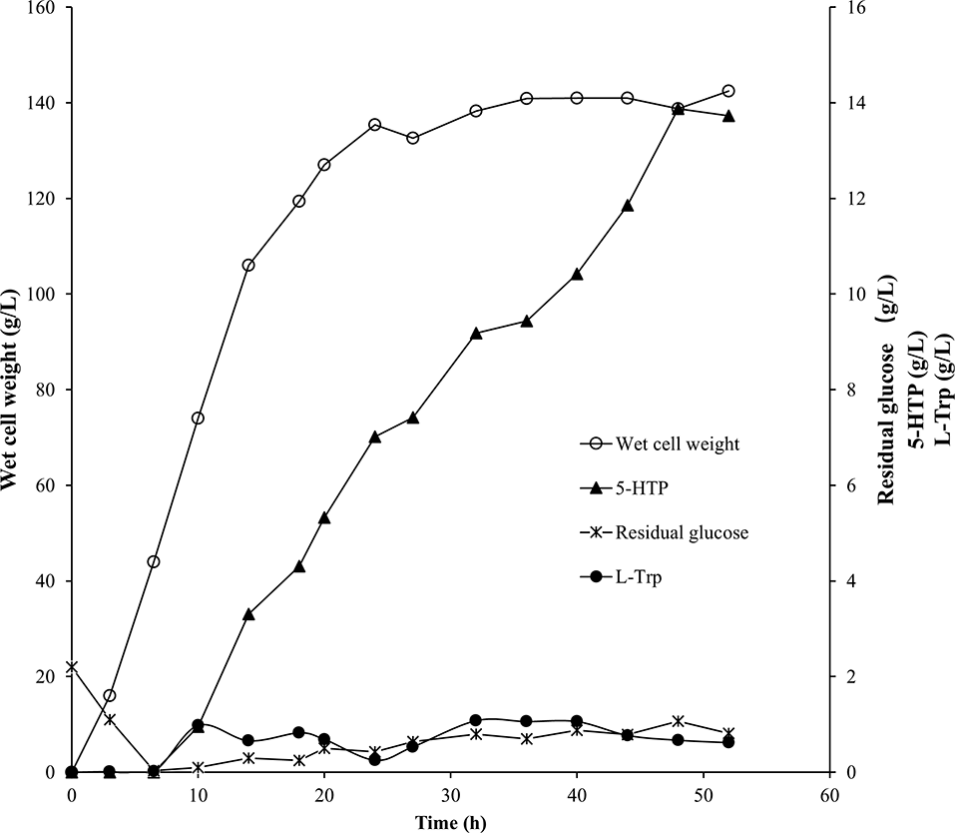
5 L-scale fed-batch biotransformation for the production of 5-HTP using strain TRP5-XC4

As shown in Fig. 5, feeding and induction commenced at 6.5 h, leading the cells to enter the exponential growth phase. The wet cell weight reached 135 g/L at 24 h, and the cells subsequently entered a steady growth phase that lasted for 28 h. Following induction with the addition of L-Trp, 5-HTP was produced at a relatively stable rate, reaching 13.9 g/L after 48 h of cultivation, which corresponds to a space-time yield of 0.29 g/L/h.

Compared to the ever-highest level in 5-HTP bio-synthesis reported by Zhang et al. (Zhang et al. 2022)(8.58 g/L production and 0.27 g/L/h space-time yield), this study demonstrated a superior 5-HTP titer of 13.9 g/L and a productivity of 0.29 g/L/h. In the context of industrial synthesis of 5-HTP, achieving higher production is crucial for enhancing equipment utilization rates, increasing the yield of single batches, and improving isolated yields, all while reducing labor costs and energy consumption.

During the 52 h of biotransformation process, approximately 25 g of exogenously added L-Trp were consumed (i.e. 122 mol), resulting in the production of about 50 g of 5-HTP (i.e. 227 mmol) from a final fermentation volume of approximately 3.6 L. The high conversion rate 186%, which exceeds 100%, can be attributed to the endogenous synthesis of L-Trp by the strain TRP5-XC4, utilizing glucose as the raw material. This phenomenon not only reduced the cost of 5-HTP biosynthesis but also underscored the importance of the “increasing income and reducing expenditure” strategy.

## Conclusions

The present study developed an L-Trp accumulating strain, designated TRP1, by blocking the degradation pathway, branching pathways, and repression system. Using strain TRP1 as the initial chassis, a hydroxylation module was screened, which employed a phenylalanine hydroxylase mutant, XcPAH^W179F^ (XC2), in conjunction with a MH4 regenerating system (CvPCD-EcFolM system) to convert L-Trp into 5-HTP. An enzyme-substrate complex of the hydroxylase and L-Trp was constructed and subjected to MD simulation, which facilitated the identification of engineering targets for combinatorial mutation. A triple mutant, XcPAH^L98I/A129K/W179F^ (XC4), was selected, enabling strain TRP1-XC4 to produce 319 mg/L of 5-HTP after 24 h of shake-flask fermentation using L-Trp as the substrate. To alleviate production inhibition, the efflux pump YddG was enhanced by inserting a strong promoter, resulting in strain TRP2-XC4, which was capable of producing 519 mg/L of 5-HTP. The production of 5-HTP was further increased to 1.81 g/L following cofactor engineering that strengthened the expression of MH4-related enzymes FolM, FolE, and FolX. Finally, strain TRP5-XC4 was utilized in a 5 L-scale fed-batch biotransformation. The titer of 5-HTP reached 13.9 g/L after 48 h of fermentation, demonstrating a space-time yield of 0.29 g/L/h, which represents the highest production and productivity record for 5-HTP biosynthesis.

## Electronic supplementary material

Below is the link to the electronic supplementary material.

Supplementary Material 1

## Data Availability

All data generated or analyzed during this study are included in this published article and its supplementary information files.

## Abbreviations

5-HTTP: 5-Hydroxytryptophan
Trp: Tryptophan; 5-HT, 5-hydroxytryptamine
MD: Molecular dynamics
TPH: Tryptophan hydroxylase
BH4: Tetrahydrobiopterin
PAH: Phenylalanine hydroxylase
AAAHs: Aromatic amino acid hydroxylases
MH4: 5,6,7,8-tetrahydromonapterin
DO: Dissolved oxygen
RMSF: Root mean square fluctuation
H2-NPt-P3: 7,8-Dihydroneopterin 3’-triphosphate; H2-MPt-P3, 7,8-Dihydromonapterin 3’-triphosphate
H2-NPt-P: 7,8-Dihydroneopterin 3’-phosphate
H2-NPt: 7,8-Dihydroneopterin
H2-MPt: 7,8-Dihydromonapterin
4a-MH4: Pterin-4a-carbinolamine
