# Supplementary material for: Engineered phenylalanine hydroxylase coupled with an effective cofactor synthesis and regeneration system for high-yield production of 5-hydroxytryptophan
Source: Bioresour Bioprocess. 2025 Mar 6;12(1):15. doi: 10.1186/s40643-025-00846-z (PMC11885754; doi:10.1186/s40643-025-00846-z)
Supplement: Supplementary file 1 — Supplementary Material 1 [file 40643_2025_846_MOESM1_ESM.docx]

**Supporting information**

**Engineered Phenylalanine Hydroxylase Coupled with an Effective Cofactor Synthesis and Regeneration System for High-Yield Production of 5-Hydroxytryptophan**

Yulin Ai ^a^, Yusong Huang ^c^, Hongru Zhao ^c^, Bingmei Su ^a, b, *^, Juan Lin ^a, b, *^

^a^ College of Biological Science and Engineering, Fuzhou University, Fuzhou, China, 350108.

^b^ Institute of Enzyme Catalysis and Synthetic Biotechnology, Fuzhou University, Fuzhou, China, 350108.

^c^ College of Chemical Engineering, Fuzhou University, Fuzhou, China, 350108.

^*^ Correspondence author: Bingmei Su, [subingmei@fzu.edu.cn](mailto:subingmei@fzu.edu.cn); Juan Lin, [ljuan@fzu.edu.cn](mailto:ljuan@fzu.edu.cn).

Contents

**Supporting tables** S3

Table S1. Primer information S3

Table S2. Plasmids used in this study S7

Table S3. Information of used strains S8

**Supporting figures** S9

Figure S1. SDS-PAGE analysis for strains S9

Figure S2. Comparison in the RMSF of the loop structures between XC2 and XC4 S10

Figure S3. Biosynthesis of MH4 in *E. coli* S11

**Tables**

**Table S1. Primer information.**

| Primer | Sequence (5’-3’) |
| --- | --- |
| XC-F | atcggatccatgaacaccgcccctcg |
| XC-CV-R | gattcatggtatatctccttcaagaattcttaaacatcaccatcacgtgaccag |
| CV-F | gaattcttgaaggagatataccatgaatctgctggaactgagctg |
| CV-EC-R | gttacggacgacgaaacagtgc |
| CV-EC-F | gaagcactgtttcgtcgtccgtaacgagctcaaggagatataccatgggtaaaacccag |
| EC-R | gtgctcgagttaacgcagatgacgacc |
| CT-F | atcggatccatgagcatcgcaatggcaac |
| CT-CV-R | gattcatggtatatctccttcaagaattcttaaatatcttcggtatctgcccaacc |
| DJ-F | atcggatccatgtgcgaacgtgataaagttttcg |
| DJ-CV-R | gattcatggtatatctccttcaagaattcttaaatgctctgatctgcttcactg |
| CV-CVDpr-F | gcactgtttcgtcgtccgtaacgagctcaaggagatataccatgaacatcgcacac |
| CVDpr-R | gtgctcgagttacagacgggtaatcactgcttc |
| CV-NfsB-F | gcactgtttcgtcgtccgtaacgagctcaaggagatataccatggatatcatttctgtc |
| NfsB-R | gtgctcgagttacacttcggttaaggtgatgttttgc |
| *tnaA*-gRNA-F | atcgatttactgaccgacaggttttagagctagaaatagca |
| *tnaA*-gRNA-R | ctgtcggtcagtaaatcgatactagtattatacctaggactg |
| *pheA/tyrA*-gRNA-F | acaaaacttcacccgatttggttttagagctagaaatagca |
| *pheA/tyrA*-gRNA-R | caaatcgggtgaagttttgtactagtattatacctaggactg |
| *trpR*-gRNA-F | cagaacagcgtcaccaggaggttttagagctagaaatagca |
| *trpR*-gRNA-R | ctcctggtgacgctgttctgactagtattatacctaggactg |
| *trpL*-gRNA-F | caattttcgtactgaaaggtgttttagagctagaaatagca |
| *trpL*-gRNA-R | acctttcagtacgaaaattgactagtattatacctaggactg |
| *yddg*-pTrc-gRNA-F | cgacaaaaagcaacgctcatgttttagagctagaaatagca |
| *yddg*-pTrc-gRNA-R | atgagcgttgctttttgtcgactagtattatacctaggactg |
| *folM*-pTrc-gRNA-F | atcccatcgttggatagcaagttttagagctagaaatagca |
| *folM*-pTrc-gRNA-R | ttgctatccaacgatgggatactagtattatacctaggactg |
| *folE*-pTrc-gRNA-F | ggcggaggcgtcacacctgcgttttagagctagaaatagca |
| *folE*-pTrc-gRNA-R | gcaggtgtgacgcctccgccactagtattatacctaggactg |
| *folX*-pTrc-gRNA-F | tttcatcctaagtaaaacaagttttagagctagaaatagca |
| *folX*-pTrc-gRNA-R | ttgttttacttaggatgaaaactagtattatacctaggactg |
| *tnaA*-U-F | ggtaagtaaccgcgcttacg |
| *tnaA*-U-R | atccttatagccactctgcagtgatccctgtgaatattacatctgc |
| *tnaA*-D-F | cagagtggctataaggatgttagcc |
| *tnaA*-D-R | gtaatgcgactggcggc |
| *pheA/tyrA*-U-F | ccatccgccaacatgtcg |
| *pheA/tyrA*-U-R | cgcgtggcttaagaggggcccccgatttgggag |
| *pheA/tyrA*-D-F | cctcttaagccacgcgagc |
| *pheA/tyrA*-D-R | aaaaacggcaccgacgg |
| *trpR*-U-F | cgatggcgattgcccgtca |
| *trpR*-U-R | tcggtgcacgatgcctttagcgggggaagcaaaatgc |
| *trpR*-D-F | aggcatcgtgcaccgaatg |
| *trpR*-D-R | cattctgcaggcatttcacgaga |
| *trpL*-U-F | gccacaaggtcataagagaacaggc |
| *trpL*-U-R | acgtaaaaagggtatccagatacccagcccgcctaa |
| *trpL*-D-F | gataccctttttacgtgaacttgcgt |
| *trpL*-D-R | cttccagcgccccaggta |
| *yddg*-pTrc-U-F | catttctggttgtggcgtgagaaaata |
| *yddg*-pTrc-U-R1 | aattgaagaaggagatatacatatgatggtaggattgattcgcgg |
| *yddg*-pTrc-U-R2 | ttgacaattaatcatccggctcgtataatgtgtggaattgaagaaggaga |
| *yddg*-pTrc-D-F | acgagccggatgattaattgtcaagttaaaaattaagctgaattttat |
| *yddg*-pTrc-D-R | aatccagcaaaccggccccttttgggca |
| *folM*-pTrc-U-F | atcgtttccgtttgcggcgcgtac |
| *folM*-pTrc-U-R | acgagccggatgattaattgtcaattatcccaccagcatccaggtcgc |
| *folM-*pTrc-D-F1 | tggaattgaagaaggagatatacatatgatgggtaaaacccagcc |
| *folM*-pTrc-D-F2 | ttgacaattaatcatccggctcgtataatgtgtggaattgaagaaggaga |
| *folM*-pTrc-D-R | cacttccggtgccagcttgc |
| *folE*-pTrc-U-F | cagccacgttattggtgcccagcagcgtttgt |
| *folE*-pTrc-U-R1 | tgtggaattgaagaaggagatatacatatgatgccatcactcagtaaagaagcg |
| *folE*-pTrc-U-R2 | ttgacaattaatcatccggctcgtataatgtgtggaattgaagaaggaga |
| *folE*-pTrc-D-F | acgagccggatgattaattgtcaatttacagcctgattattgtgcgtgaggcggcg |
| *folE*-pTrc-D-R | cccacggcggttgcgtggcattaagataaaag |
| *folX*-pTrc-U-F | attccggcaattgttgatcattctcctg |
| *folX*-pTrc-U-R | gccggatgattaattgtcaatacacgagaacctgtttctgttaactatccg |
| *folX-*pTrc-D-F1 | ttgacaattaatcatccggctcgtataatgtgtggaattgaagaaggaga |
| *folX-*pTrc-D-F2 | tgtggaattgaagaaggagatatacatatgatggcacaacctgccgctattattcgt |
| *folX-*pTrc-D-R | ttgccatagtgtcacccgagggccg |
| XcPAH-W179F-F | ctgtatttttataccgtggaatttggcctgattgcaaccc |
| XcPAH-W179F-R | ggtataaaaatacaggcgggtcagattttgcagt |
| XcPAH-W179L-F | gacccgcctgtatctgtataccgtggaatttggc |
| XcPAH-W179L-R | gccaaattccacggtatacagatacaggcgggtc |
| XcPAH-W179M-F | gacccgcctgtatatgtataccgtggaatttggc |
| XcPAH-W179M-R | gccaaattccacggtatacatatacaggcgggtc |
| XcPAH-W179Y-F | gacccgcctgtattattataccgtggaatttggc |
| XcPAH-W179Y-R | gccaaattccacggtataataatacaggcgggtc |
| XcPAH-W179I-F | gacccgcctgtatatttataccgtggaatttggc |
| XcPAH-W179I-R | gccaaattccacggtataaatatacaggcgggtc |
| XcPAH-L98I-F | gtgttgaaggtattctgccggaactggatt |
| XcPAH-L98I-R | gttccggcagaataccttcaacacccacc |
| XcPAH-L98F-F | ggtgttgaaggttttctgccggaactggatt |
| XcPAH-L98F-R | cggcagaaaaccttcaacacccaccag |
| XcPAH-L98Y-F | gggtgttgaaggttatctgccggaactggattt |
| XcPAH-L98Y-R | gttccggcagataaccttcaacacccaccag |
| XcPAH-L98V-F | ttgaaggtgtgctgccggaactgg |
| XcPAH-L98V-R | cggcagcacaccttcaacacccac |
| XcPAH-L99I-F | ggtctgattccggaactggattttttt |
| XcPAH-L99I-R | ttccggaatcagaccttcaacacccacca |
| XcPAH-L99V-F | ggtctggtgccggaactggatttttttga |
| XcPAH-L99V-R | ttccggcaccagaccttcaacacccacc |
| XcPAH-Y178F-F | cgcctgtttttttataccgtggaatttggcct |
| XcPAH-Y178F-R | caaattccacggtataaaaaaacaggcgggtcagattt |
| XcPAH-Y178H-F | cgcctgcatttttataccgtggaatttggcct |
| XcPAH-Y178H-R | caaattccacggtataaaaatgcaggcgggtcagattt |
| XcPAH-I233V-F | cgttatcgcgtggatagttttcagaaaacctacttcg |
| XcPAH-I233V-R | tgaaaactatccacgcgataacgggtacgcatcacac |
| XcPAH-I233A-F | ccgttatcgcgcggatagttttcagaaaacctacttcgt |
| XcPAH-I233A-R | tctgaaaactatccgcgcgataacgggtacgcatcaca |
| XcPAH-I233L-F | ccgttatcgcctggatagttttcagaaaacctacttcgt |
| XcPAH-I233L-R | ttctgaaaactatccaggcgataacgggtacgcat |
| XcPAH-A129K-F | cggatcagattgattatattaaagaaccggatctgtttcatgatctg |
| XcPAH-A129K-R | acagatccggttctttaatataatcaatctgatccggacgac |
| XcPAH-A129N-F | cagattgattatattaacgaaccggatctgtttcatgatctg |
| XcPAH-A129N-R | cagatccggttcgttaatataatcaatctgatccggac |
| XcPAH-A129P-F | cagattgattatattccggaaccggatctgtttcatgatctgt |
| XcPAH-A129P-R | cagatccggttccggaatataatcaatctgatccggac |
| XcPAH-A129R-F | cagattgattatattcgcgaaccggatctgtttcatgatctg |
| XcPAH-A129R-R | gatccggttcgcgaatataatcaatctgatccggacg |
| XcPAH-A129T-F | cggatcagattgataccattaaagaaccggatctgtttcatgatctg |
| XcPAH-A129T-R | agatccggttcggtaatataatcaatctgatcc |
| XcPAH-A129E-F | cagattgattatattgaagaaccggatctgtttcatgatctg |
| XcPAH-A129E-R | cagatccggttcttcaatataatcaatctgatccggacgac |
| XcPAH-A129G-F | cggatcagattgattatattggcgaaccggatctgtttcatgatctg |
| XcPAH-A129G-R | catgaaacagatccggttcgccaatataatcaatctgatccggac |
| XcPAH-Y127F-F | cggatcagattgattttattaaagaaccggatctgtttcatgatctg |
| XcPAH-Y127F-R | acagatccggttctttaataaaatcaatctgatccggacgac |
| XcPAH-Y127H-F | cggatcagattgatcatattaaagaaccggatctgtttcatgatctg |
| XcPAH-Y127H-R | acagatccggttctttaatatgatcaatctgatccggacgac |
| XcPAH-F184Y-F | gtggaatatggcctgattgcaaccccgc |
| XcPAH-F184Y-R | caggccatattccacggtataccaataca |
| XcPAH-A198S-F | gcgtatttatggcagcggcattgtgagcagt |
| XcPAH-A198S-R | gctcacaatgccgctgccataaatacgcaga |
| XcPAH-A198G-F | atttatggcggcggcattgtgagcagt |
| XcPAH-A198G-R | cacaatgccgccgccataaatacgcaga |

**Table S2.** **Plasmids used in this study.**

| Plasmid | Description |
| --- | --- |
| pET30a | Expressing plasmid, Kan^+^ |
| pXC1 | pET30a-*xcpah-**cvpcd-ecfolm* |
| pCT1 | pET30a-*ctpah-cvpcd-ecfolm* |
| pDJ1 | pET30a-*djtph-cvpcd-cvdpr* |
| pDJ2 | pET30a-*djtph-cvpcd-ecnfsb* |
| pXC2 | pET30a-*xcpah*^W179F^-*cvpcd-ecfolm* |
| pXC3 | pET30a-*xcpah*^W179F/L98I^-*cvpcd-ecfolm* |
| pXC4 | pET30a-*xcpah*^W179F/L98I/A129K^-*cvpcd-ecfolm* |
| pEcCas | Expression of Cas9 and recombinase, Kan^+^ |
| pTargetF | Transcription of gRNA scaffold, Str^+^ |
| pTargetF-g-*tnaA* | Targeting plasmid for gene *tnaA* |
| pTargetF-g-*pheA*/*tyrA* | Targeting plasmid for gene *pheA*/*tyrA* |
| pTargetF-g-*trpR* | Targeting plasmid for gene *trpR* |
| pTargetF-g-*trpL* | Targeting plasmid for gene *trpL* |
| pTargetF-g-*ydd*G | Targeting plasmid for gene *ydd*G |
| pTargetF-g-*fol*M | Targeting plasmid for gene *fol*M |
| pTargetF-g-*fol*E | Targeting plasmid for gene *fol*E |
| pTargetF-g-*fol*X | Targeting plasmid for gene *fol*X |

**Table S3. Information of used strains.**

| Strain | Features | Source |
| --- | --- | --- |
| *E.* *coli* DH5α | Cloning host | This lab |
| *E.* *coli* BL21(DE3) | Wildtype chassis cells | This lab |
| TRP1 | *E.* *coli* BL21(DE3) (Δ*tnaA*, Δ*pheA*, Δ*tyrA*, Δ*trpR*, Δ*trpL*) | This study |
| TRP1-XC1 | TRP1 harboring pXC1 | This study |
| TRP1-CT1 | TRP1 harboring pCT1 | This study |
| TRP1-DJ1 | TRP1 harboring pDJ1 | This study |
| TRP1-DJ2 | TRP1 harboring pDJ2 | This study |
| TRP1-XC2 | TRP1 harboring pXC2 | This study |
| TRP1-XC3 | TRP1 harboring pXC3 | This study |
| TRP1-XC4 | TRP1 harboring pXC4 | This study |
| TRP2 | *E.* *coli* BL21(DE3) (Δ*tnaA*, Δ*pheA*, Δ*tyrA*, Δ*trpR*, Δ*trpL*, P_trc_-*yddG*) | This study |
| TRP2-XC4 | TRP2 harboring pXC4 | This study |
| TRP3 | *E.* *coli* BL21(DE3) (Δ*tnaA*, Δ*pheA*, Δ*tyrA*, Δ*trpR*, Δ*trpL*, P_trc_-*yddG*, P_trc_-*folM*) | This study |
| TRP3-XC4 | TRP3 harboring pXC4 | This study |
| TRP4 | *E.* *coli* BL21(DE3) (Δ*tnaA*, Δ*pheA*, Δ*tyrA*, Δ*trpR*, Δ*trpL*, P_trc_-*yddG*, P_trc_-*folM*, P_trc_-*folE*) | This study |
| TRP4-XC4 | TRP4 harboring pXC4 | This study |
| TRP5 | *E.* *coli* BL21(DE3) (Δ*tnaA*, Δ*pheA*, Δ*tyrA*, Δ*trpR*, Δ*trpL*, P_trc_-*yddG*, P_trc_-*folM*, P_trc_-*folE*, P_trc_-*folX*) | This study |
| TRP5-XC4 | TRP5 harboring pXC4 | This study |

**Figures**


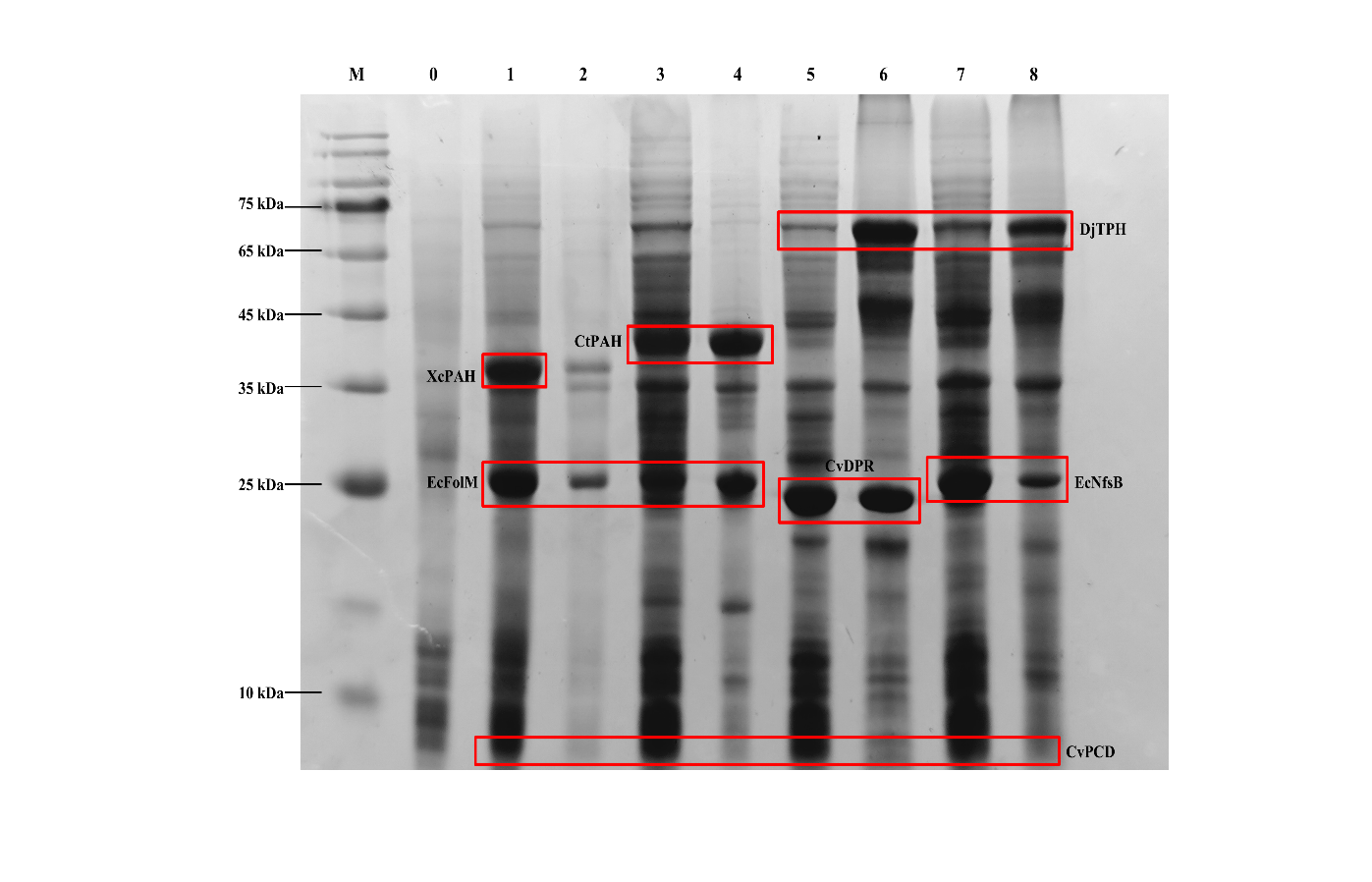


**Figure S1.** SDS-PAGE analysis for strains. Line 0, cells carrying pET30a; Line 1 and 2, supernatant and precipitation of strain TRP1-XC1; Line 3 and 4, supernatant and precipitation of strain TRP1-CT1; Line 5 and 6, supernatant and precipitation of strain TRP1-DJ1; Line 7 and 8, supernatant and precipitation of strain TRP1-DJ2.


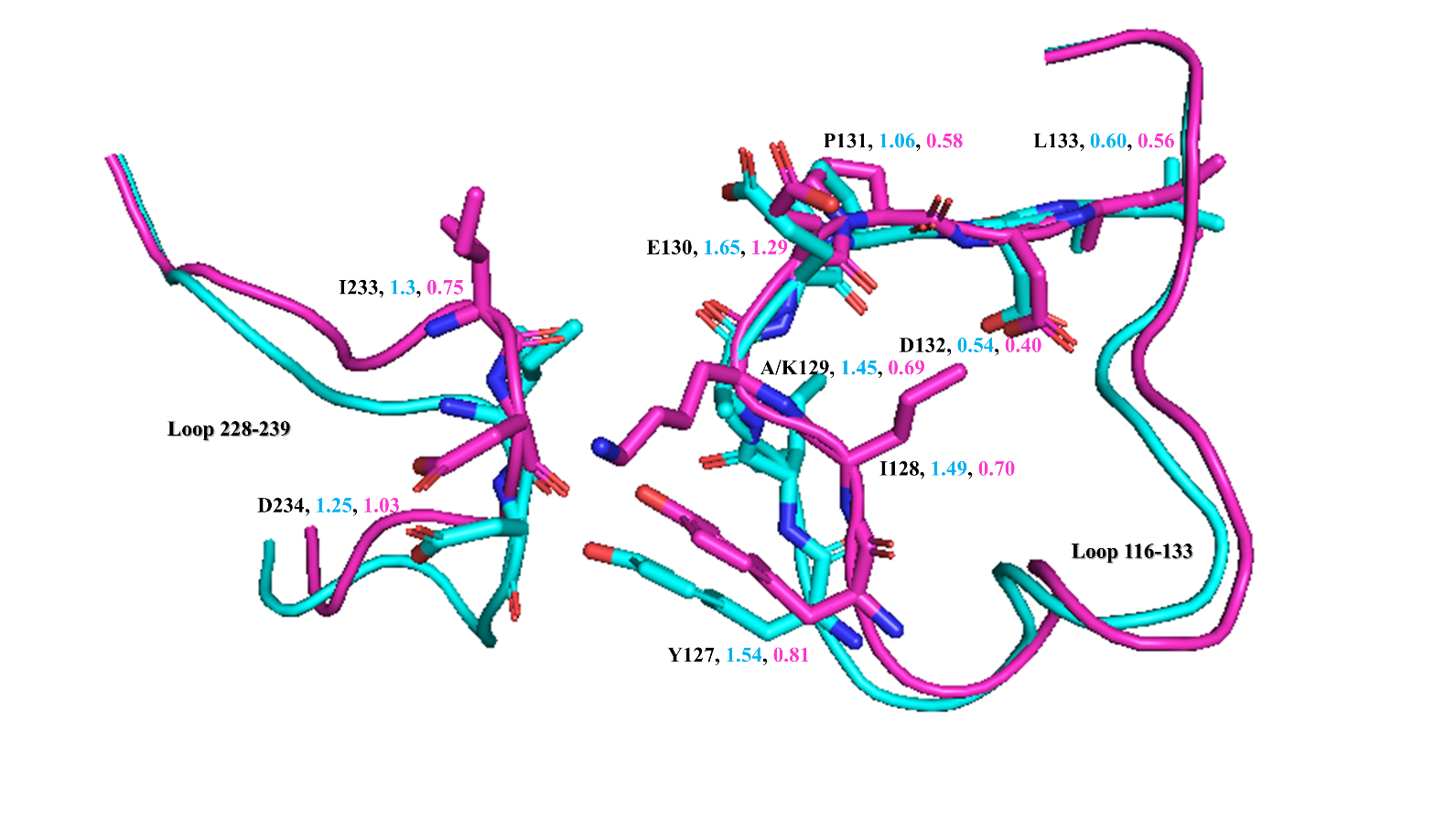


**Figure S2.** Comparison in the RMSF of the loop structures between XC2 (cyans) and XC4 (pink).


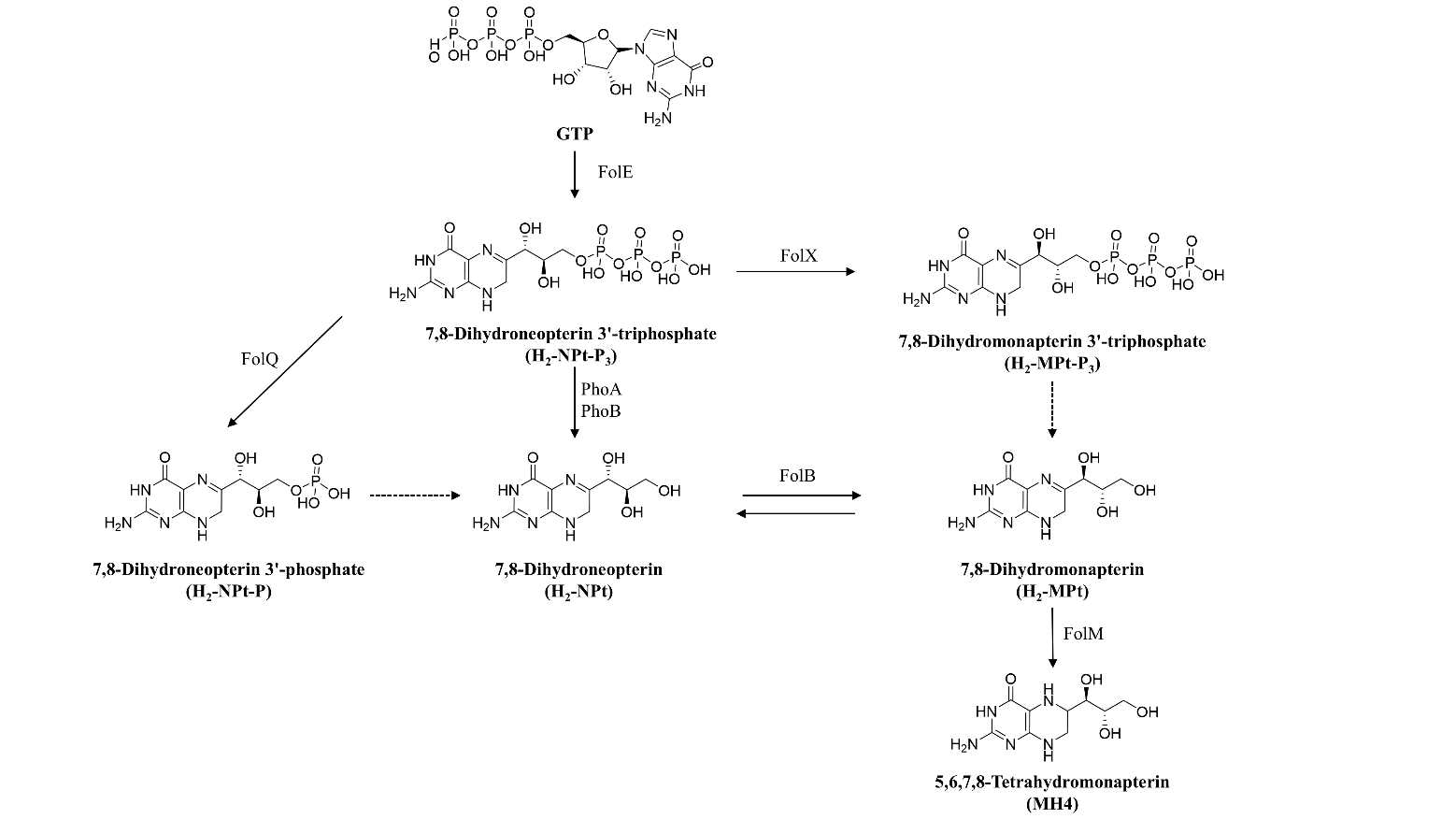


**Figure S3.** Biosynthesis of MH4 in *E. coli*.
